# Genetic Traces in Autism Spectrum Disorders: A Whole Exome Sequencing Study from Türkiye

**DOI:** 10.3390/genes17020249

**Published:** 2026-02-23

**Authors:** Gülsüm Kayhan, Ahmet Ozaslan, Elvan Işeri, Esra Guney, Hasan Huseyin Kazan, Dicle Buyuktaskin, Muhammed Fatih Mulayim, Mehmet Ali Ergun, Ferda Emriye Percin

**Affiliations:** 1 Department of Medical Genetics, Gazi University Hospital, Gazi University, 06560 Ankara, Türkiye; dr.mfmulayim@gmail.com (M.F.M.); maliergun@gmail.com (M.A.E.); ferdaep@yahoo.com (F.E.P.); 2 Department of Child and Adolescent Psychiatry, Faculty of Medicine, Gazi University, 06560 Ankara, Türkiye; drahmetozaslan@yahoo.com (A.O.); ekaracan@gazi.edu.tr (E.I.); esraguney@gazi.edu.tr (E.G.); diclebuyuktaskin@gmail.com (D.B.); 3 Department of Medical Biology, Gulhane Faculty of Medicine, University of Health Sciences, 06200 Ankara, Türkiye; hasanhuseyinkazan@gmail.com

**Keywords:** autism spectrum disorders, whole exome sequencing, molecular diagnosis

## Abstract

Background: Autism spectrum disorders (ASDs) are defined as a large spectrum of phenotypes whose basic definition is deficiency in social interactions, particularly during pediatric stages. Through clinical evaluations, it would be challenging to diagnose since the symptoms may be disregarded or controversial. Hence, molecular approaches could be powerful for differential and certain diagnosis. Moreover, considering the possible genetic complexity of the disease, the rates of molecular diagnosis remain insufficient. Nevertheless, the number of newly identified ASD-monogenic inheritance relationships is escalating daily. This underscores the increasing importance of comprehensive molecular tests, such as whole exome sequencing (WES), which encompass all relevant genes. Furthermore, reporting population-specific variants is critical to validate already listed ones and decipher novel ones. In the present study, we aimed to document the disease-related variants in Turkish patients with ASD. Methods: This study evaluated the WES outcomes of 75 ASD patients with normal results in Fragile X testing, cytogenetic analysis, and molecular karyotyping. All patients were diagnosed with ASD based on the criteria from the *Diagnostic and Statistical Manual of Mental Disorders, 5th Edition* (DSM-5). Results: The average age of the participants was 8.2 (±5.0) years. A higher percentage of the participants was male (73.3%) compared with female (26.7%). Eighteen patients (24%) had pathogenic or likely pathogenic (LP) variants, while 34 (45.3%) exhibited variants of unknown significance (VUS). In 30.7% of the cases, no clinically relevant variants were found. The *MECP2* gene was most frequently affected, followed by *EP300* and *PTEN*. Additionally, four patients carried novel de novo missense variants in the *KMT2C*, *MECP2*, *PTEN*, and *TRRAP* genes. Conclusions: Genetic diagnosis of ASD would be useful for confirming the underlying etiologies, devising personalized therapeutic strategies, and offering family counseling. Although WES has been employed in ASD patients for an extended period, the identification of gene and variant spectra across diverse cohorts and the discovery of novel variants continues to hold significant scientific importance.

## 1. Introduction

Autism spectrum disorders (ASDs) are a group of heterogeneous neurodevelopmental disorders primarily characterized by impaired social interactions and repetitive behaviors. The prevalence of ASD in a population is relatively high, ranging from 1 to 2% [1,2]. As a broad spectrum of psychiatric disorders, the diagnosis of ASDs can be challenging. The American Psychiatric Association (2013) defined several criteria for diagnosis, including assessment of eye contact, social relationships, speech delays or difficulties, and stereotyped and repetitive behaviors [3]. However, early reports emphasizing overlapping or ASD-related phenotypes such as mental retardation, seizures, Asperger syndrome, Fragile X syndrome, and phenylketonuria—with diverse ratios in patients—have highlighted the challenges in the differential diagnosis of ASDs [4,5].

In some cases, the differential diagnosis of ASD would be critical for managing primary or secondary phenotypes such as intellectual disability [6]. Nevertheless, a so-called gold-standard diagnostic approach is not possible for such a complex disorder through conventional psychiatric evaluations, since patients should be followed throughout their lifespan to assess disease severity [7]. In this context, genetic tools could be beneficial in reducing subjectivity and concluding differential diagnoses [8]. Estimates suggest that ASD results from genetic factors in about 90% of cases; however, twin studies indicate that this ratio is approximately 50% when considering environmental factors [9,10].

Aside from the clinical presentation, the genetic background of ASD is remarkably complex. Therefore, the rates of genetic diagnosis remain low, particularly in isolated ASD cases without additional findings [8,9,10]. Copy number alterations, a genetic mechanism of ASD, can be identified through molecular karyotyping, which is often the first-line test at many centers. However, copy number alterations account for only a small fraction of the molecular causes of ASD [11]. Most cases follow a monogenic inheritance pattern, with many related genes identified. A significant number of these cases are reported as autosomal-dominant [8], though recessive and polygenic inheritance patterns are also observed in ASD [12,13]. Thus, genetic screening for ASD requires a comprehensive genetic tool for differential diagnosis [14]. In the era of DNA sequencing technologies in clinical genetics, whole exome sequencing (WES) is a powerful tool that covers all exons and exon-intron boundaries throughout the genome. Hence, WES can enhance screening for previously reported variants across all genes and allow exploration of novel variants or genes linked to ASD [15]. Still, one of the major concerns in WES analysis is the evaluation of variants of unknown significance (VUSs). Reporting VUSs can be challenging, as it may mislead patient management or lead to the omission of other critical variants. Therefore, VUSs should be re-evaluated over time, as recent studies suggest [16], which further necessitates phenotype-genotype correlation-based studies.

Accordingly, several cohort studies using WES for the molecular diagnosis of ASD in various populations have been conducted, and novel associations have been reported [17,18,19,20,21]. Nonetheless, such population-based cohort studies are still needed to confirm the association between ASD and the reported genes, identify new linked variants or genes, and demonstrate any possible population differences.

In the present study, we re-evaluated the WES results of 75 patients for whom autism was questioned. The study emphasized the efficacy of the WES in accordance with genetic algorithms for the molecular and differential diagnosis of autism by reporting a high ratio of pathogenic and likely pathogenic (LP) variants in the Turkish population.

## 2. Materials and Methods

### 2.1. Ethics

The present study was approved by the Gazi University Ethics Committee under approval number 2025-754.

### 2.2. Participants

This study retrospectively evaluated the WES results of 75 Turkish children with ASD. The patients enrolled in the Child and Adolescent Psychiatry Department of Gazi University Hospital were routinely directed to the Department of Medical Genetics of Gazi University Hospital in Ankara between 2017 and 2024 for molecular and differential diagnosis.

The participants had to meet certain criteria to be included in the study. They had to be between 1 and 18 years old and have an ASD diagnosis that met the *Diagnostic and Statistical Manual of Mental Disorders, 5th Edition* (DSM-5) criteria. Two child and adolescent psychiatrists confirmed this diagnosis, with at least one having more than 5 years of experience treating children with ASD. The study excluded children and adolescents with ASD who did not meet these criteria and those whose genetic tests were incomplete. It also excluded patients with chromosomal anomalies, microdeletions or duplications, or Fragile X syndrome. Before the study, all participants provided their verbal and written consent.

### 2.3. Whole Exome Sequencing and Analysis

WES was performed using the genomic DNA isolated from the participants’ peripheral blood samples. For sequencing, libraries were prepared using the Illumina DNA Prep and Exome 2.5 Enrichment kit (Illumina Inc., San Diego, CA, USA) in some patients and the Twist Exome 2.0 kit (Twist Bioscience, South San Francisco, CA, USA) in others. The NovaSeq 6000 Sequencing System (Illumina Inc., San Diego, CA, USA) was employed for the sequencing of prepared libraries, following the supplier’s routine protocol. Each sample’s mean read depth was 20, and the ≥20× depth percentage was 98%.

**Figure 1:**
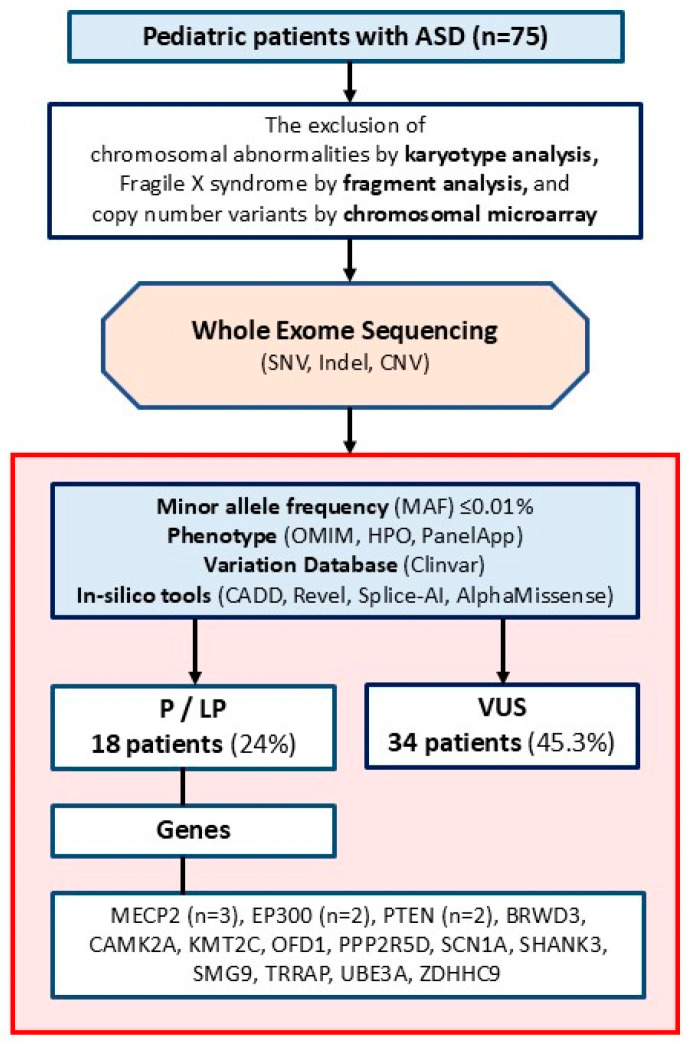
The flowchart of the study.

Sequencing data from certain patients were analyzed using version 8.0 of the Genomize SEQ Platform (Genomize, Türkiye), whereas data from other patients were analyzed with version 3.3 of the Ilyome software (Ilyome, Türkiye). On these platforms, raw fastq files were aligned to the human reference genome (GRCh38), converted to binary alignment and map (bam) files, and then converted to variant call format (vcf) files. Using the same platforms, single nucleotide variants (SNVs) and copy number variants (CNVs) were filtered for a minor allele frequency (MAF) of <0.01% in standard databases (GnomAD global). A phenotype filter was then applied using genes from the Human Phenotype Ontology (HPO) database related to nervous system abnormalities (HP:0000707), OMIM-morbid genes, and genes from the PanelApp intellectual disability panel, including known ASD genes. Deleterious variants that are homozygous or compound heterozygous were also reexamined without using any phenotypic filter. The remaining variants were prioritized following the standards of the American College of Medical Genetics and Genomics [22], considering the variant type (null, in-frame, missense, intronic, etc.), disease mechanism, literature reports or data from variant databases, results from functional analyses, population frequency, variant location, variant position in recessive diseases, predictions from in silico tools about protein effects, and the connection between the gene and the phenotype. In silico analysis was conducted using CADD, Revel, Splice-AI, and AlphaMissense. The thresholds for pathogenicity assessment were set at ≥20 for CADD, ≥0.6 for Revel, ≥0.2 for Splice-AI, and ≥0.7 for AlphaMissense. Pathogenic and LP variants, as well as VUSs, were evaluated. When selecting VUSs, we included missense and intronic variants within the ±10 splicing region (Figure 1).

Segregation analysis of the variants was conducted using Sanger sequencing with the BigDye™ Terminator v3.1 Cycle Sequencing Kit (Thermo Scientific, Waltham, PA, USA) and the Applied Biosystems 3130 Genetic Analyzer (Thermo Scientific, USA). Regarding de novo variants, the parent–child relationship was confirmed by analyzing supplementary rare variants within the WES dataset.

## 3. Results

### 3.1. Clinical Findings

The study included 75 children with ASD, as determined by the primary clinical evaluation conducted by the Child and Adolescent Psychiatry Department. The mean age of the patients was 8.2 (±5.0) years. Of this cohort, 20 patients (26.7%) were female, while the remaining 55 (73.3%) were male (Table 1 and Table 2).

### 3.2. Molecular Findings

In 75 cases with ASD, 18 patients (24%) had pathogenic or LP variants that explained the genetic background of the autism-related phenotypes (Table 1). One of these patients had two different LP variants in the SMG9 nonsense-mediated mRNA decay factor (*SMG9*) gene, inherited separately from parents in a compound heterozygous manner and constituting an autosomal recessive syndrome. Therefore, the total number of pathogenic or LP variants in the cohort was 19. Among the pathogenic group, methyl-CpG binding protein 2 (*MECP2*) was the most frequently affected gene, observed in three (16.7%) independent patients. This gene was followed by the E1A binding protein P300 (*EP300*) and phosphatase and tensin homolog (*PTEN*) genes, each of which were affected in two patients.

Among the patients with pathogenic or LP variants, three patients with additional non-neurological findings were diagnosed with *EP300*-associated Rubinstein–Taybi syndrome (P5), ubiquitin protein ligase E3A (*UBE3A*)-associated Angelman syndrome (P62), and *OFD1*-associated ciliopathy syndrome (P71; Table 1). Notably, two patients (2.7%; P5 and P75) had a dual molecular diagnosis.

Eleven novel pathogenic or LP variants were detected in 10 patients. Four of these were missense variants classified as LP because they were found de novo in the lysine methyltransferase 2C (*KMT2C*), *PTEN*, *MECP2*, and transformation/transcription domain-associated protein (*TRRAP*) genes. The remaining six were nonsense variants, and one was a missense variant detected in the trans position alongside a nonsense variant in the *SMG9* gene associated with recessive inheritance.

Moreover, 34 (45.3%) patients had at least one VUS (Table 2). A patient (P66) in this group had a compound heterozygous variant in the HECT and RLD domain containing an E3 ubiquitin protein ligase 2 (*HERC2*) gene associated with an autosomal recessive disorder. Importantly, one of the patients (P67) had two VUSs in lysine methyltransferase 2B (*KMT2B*) and cyclin-dependent kinase 8 (*CDK8*) inherited from the affected mother.

No clinically relevant variants were detected for the remaining patients (23 out of 75; 30.7%).

## 4. Discussion

The present study documented the results of WES in 75 Turkish patients with autism-related phenotypes. Accordingly, 18 patients (24%) were molecularly diagnosed with ASD, emphasizing the capability of WES in diagnosing autism within the Turkish population.

Autism could be characterized by difficulties in communication and social interaction and repetitive or restricted interests and behaviors. It may also include additional features such as learning difficulties. By this definition, clinically diagnosing early-onset symptoms of pediatric cases would be challenging. Thus, given the high prevalence of the disorder (1% worldwide in 2021) [23], a certain diagnosis requires alternative approaches in addition to clinical observations of patients [24,25]. Here, genetic screening could serve as a beneficial strategy for the differential diagnosis of autism-related phenotypes [26].

For genetic testing of autism-related phenotypes, a chromosomal microarray for CNVs and fragment analysis for detecting Fragile X syndrome for male patients in particular are applicable [27]. Additionally, gene panels targeting previously reported and limited autism-linked genes are eligible [28,29,30]. To date, more than 100 risk genes have been reported in autism phenotypes [31]; however, considering the complex neuromolecular basis of autism, more candidate genes could be identified, which is a limitation of the gene panels. Therefore, WES could be a strong approach for routine diagnosis and deciphering novel associated genes or variants [32].

In the present study, the mean age of the patients was 8.2 (±5.0) years, which correlated with the literature [33]. In the cohort, the number of males was markedly higher than that of females (73.3% vs. 26.7%). The vulnerability of males to ASD has unequivocally been demonstrated and explained by the number of chromosome X and sex hormones [34], which confirms the findings of the present study.

The molecular diagnostic yield of autism drastically varies depending on the genetic testing methods. For gene panels, for example, diagnostic yields have been shown to range from 0.22 to 10.02% based on the selected genes in the panel [30]. For WES, it was 8.4% for 258 Canadian patients [35], 13.8% for 457 American patients [36], and 29.7% for 101 Indian patients [37]. The diagnostic yield of WES (percent of pathogenic and LP variants) in the present study was 24%, which is relatively high compared with other populations. However, the yield was low in comparison with a Turkish cohort of 22 patients, 11 (50%) of whom were molecularly diagnosed [38]. Overall, the present and previous studies emphasize the effectiveness of WES in the Turkish population.

Among pathogenic or LP variants, mutations in the X chromosome-localized *MECP2* gene were identified in three patients, representing 4% of all cases and 17% of those with pathogenic variants (Table 1). The phenotypic spectrum associated with *MECP2* ranges from classic or variant Rett syndrome in women to milder learning disabilities. In males, Rett syndrome-related variants typically cause neonatal encephalopathy, which is often lethal, whereas non-Rett syndrome pathogenic variants cause syndromic or nonsyndromic intellectual disability with or without epilepsy (intellectual developmental disorder, X-linked, syndromic 13 (MRXS13; OMIM #300055)). Truncating variants predicted to trigger nonsense-mediated mRNA decay (NMD) are typically linked to Rett syndrome. While some missense variants can also lead to Rett syndrome, others may cause MRXS13 [39]. In this study, a hemizygous frameshift variant (Val392CysfsTer23) in the *MECP2* gene was identified in a 10-year-old male (P53) with ASD and drug-resistant epilepsy. The fact that the frameshift variant in the patient caused MRXS13 rather than Rett syndrome may be due to its location, which is close to the end of the protein’s domain. Indeed, late-truncating variants have been associated with a milder phenotype [39]. Another male (P54), age 19 and carrying a novel de novo missense variant (Gly175Glu) in MECP2, presented with ASD, intellectual disability, epilepsy, and microcephaly (−3.8 SD). The variant, not found in the population data, is located in the methyl-CpG-binding domain (MBD) of the protein, which binds double-stranded DNA. Mutations in this domain, where pathogen variants are most common, have been shown to impair protein stability. However, a clear genotype–phenotype relationship has not been established for variants in this domain [40]. The Gly175Trp variant at the same codon was listed as LP in ClinVar (Accession: VCV000498088.5), but no phenotypic information was available in the database. Our patient’s phenotype aligned with MRXS13. An 8-year-old female (P55) with ASD, developmental regression, and epilepsy had a de novo truncating heterozygous *MECP2* mutation (Arg306Ter), consistent with Rett syndrome. Regarding the phenotype, it is likely that this earlier nonsense variant led to premature protein termination and thus to NMD. However, further functional studies are needed to clarify these predictions. Multiple de novo mutations in *MECP2* have previously been reported [41], indicating that this gene is susceptible to variation and should be prioritized when analyzing autism patients from the Turkish population.

The second most frequently varied genes were *EP300* and *PTEN*, which were each found in two patients. Although macrocephaly is often observed in *PTEN*-associated ASD [42], it was present in one patient (P9) but not in another (P8). The Tyr68His variant in P8, who lacked macrocephaly, was inherited from a father with epilepsy and major depression. This variant, primarily associated with *PTEN*-hamartoma tumor syndrome [43,44,45], was also found in a patient with macrocephaly and ASD and is a known pathogenic variant [46]. The Asn48Asp variant detected at P9 was previously unreported and not found in the population database. The Asn48Lys variant at the same codon has been identified in many individuals with *PTEN*-hamartoma tumor syndrome [47,48,49]. Although the asparagine residue is highly conserved, it was replaced by aspartic acid, which has incredibly similar characteristics. However, this alteration is predicted to be deleterious using in silico prediction tools. This variant, detected de novo for P9 and clinically consistent with *PTEN*-related macrocephaly and autism syndrome, was interpreted as LP.

One patient (P4) with an *EP300* variant showed only ASD features without dysmorphic traits. The identified nonsense variant was in exon 31, the last exon, consistent with the phenotype. Similar null variants in ClinVar have been reported downstream of this variant, and they are classified as pathogenic. Since null variants in the previous exon typically do not trigger NMD [50], this variant was considered LP, supporting a diagnosis of Menke-Hennekam syndrome 2. The other patient (P5), with a nonsense mutation in exon 2 of *EP300*, presented with ASD and typical Rubinstein–Taybi syndrome features, including microcephaly, short stature, a beaked nose, and broad thumbs and toes. Additionally, a 2.5 Mb duplication at 22q11.2 (hg19:22q11.21(18,900,442–21,440,514)x3) was identified via chromosomal microarray analysis. Phenotypic expression of 22q11.2 duplications varies, and some carriers may be asymptomatic. In addition, this syndrome may present an intellectual disability or learning disability, hypotonia, microcephaly, delayed psychomotor development, behavioral problems, seizures, congenital heart anomalies, velopharyngeal insufficiency, and growth retardation. This duplication may have contributed to the patient’s microcephaly and neurological findings. Seventy percent of this phenotype is inherited from a mildly affected or normal parent [51]; nonetheless, parental study could not be performed for this duplication in our patient.

Four patients were identified with novel de novo missense variants in the KMT2C, PTEN, MECP2, and TRRAP genes. These variants are likely disease-causing and can assist geneticists in further assessment. Variants in *MECP2* and *PTEN* were discussed above. In a 4-year-old male with ASD, a de novo missense change (Pro2217Leu) in *KMT2C* was identified. Mutations in *KMT2C* are associated with Kleefstra syndrome 2, which involves intellectual disability, developmental delay, and often autistic features [52]. This variant is absent from population databases, located in exon 36 of the canonical transcript, and lies outside the known functional domains. No pathogenic or LP missense variants in this exon have been reported in ClinVar. The affected codon is highly conserved and predicted to be deleterious (CADD: 20.2), uncertain (Revel: 0.34), or benign (AlphaMissense: 0.1) by in silico tools. It was classified as LP because it was de novo and showed no clinical inconsistencies. In another case, a novel missense variant (Arg960Gln) in *TRRAP* was identified in P13, who has ASD, an intellectual disability, and microcephaly. Pathogenic variants in *TRRAP* cause developmental delays with or without dysmorphic features and autism (OMIM #603015). This variant is absent from population databases and lies outside the known functional domains, with the protein region lacking homologs across species. The CADD score was 13.95 (likely benign), the Revel score was 0.51 (uncertain significance), and the AlphaMissense score was 0.92 (potentially deleterious). This de novo variant was classified as LP. Given current understanding limitations, further research is needed to clarify the structural and functional roles of these genes.

The missense Arg104Trp variant was detected in the *CAMK2A* gene, which is associated with intellectual developmental disorder (OMIM #300659), in P3. Behavioral abnormalities, including autistic features, have been reported in this syndrome [53]. Our patient also had bilateral congenital hearing loss (HL) along with ASD, although HL had not been previously reported in this condition. No variant linked to hearing loss was identified in the WES analysis. The same variant (Arg104Trp) has been classified as LP by a center in ClinVar, and hearing loss has not been reported among the patient’s findings (Accession: SCV007095856.1). Still, additional patient data is necessary to determine whether HL occurs within this syndrome.

An LP variant in the *OFD1* gene was identified in 7-year-old P71, who had ASD, speech delay, and postaxial polydactyly. The *OFD1* gene is associated with ciliopathies such as orofaciodigital syndrome (OFDS), characterized by oral, facial, digital, and neurological symptoms, and Joubert syndrome (JTS), which features visual impairment, neurological issues, and polydactyly [54,55]. The patient exhibited no oral signs of OFDS. However, without a cranial MRI, a definitive diagnosis of JTS could not be made. Therefore, the condition was generally classified as a ciliopathy associated with *OFD1*.

In this cohort, a high number of VUSs were identified, with 45 in 34 patients (45.3%). While these variants alone could not establish a definitive molecular diagnosis, their documentation is crucial. They may be reclassified in the future research—such as additional cohort studies, functional analyses, or advances in bioinformatics analyses [56]—and may guide medical geneticists. A limitation of the study was that parental testing was only feasible in five of the patients.

A homozygous VUS in the *CEP41* gene, linked to autosomal recessive JTS, was identified in 14-year-old P72, who exhibited ASD, developmental delay, microcephaly (−5.8 SD), spasticity, visual impairments, and bilateral frontotemporal atrophy observed upon MRI at 3.5 years of age. Although this variant, rarely reported in the population and only in heterozygous form, might be associated with the patient’s clinical features, it was classified as a VUS because the brain MRI obtained at 3.5 years of age did not show signs specific to JTS, such as the molar tooth sign, and no later-age brain MRI for the patient was available.

In P75, a homozygous VUS was detected in the *C12ORF57* gene, which is associated with Temtamy syndrome. He exhibited developmental delays, EEG abnormalities, and bilateral congenital glaucoma, along with ASD. Although this patient did not possess a clear pathogenic variant linked to ASD, he had compound heterozygous pathogenic variants in the trans position in the cytochrome P450 family 1 subfamily B member 1 (*CYP1B1*) gene, which accounted for the glaucoma (Table 2). These results in this patient once again demonstrate that, as with all genetic diseases, dual diagnosis should always be considered in autism patients with additional findings.

Another limitation of the study is that although WES was proposed to identify novel ASD-related genes, the small sample size and the absence of a healthy control group limited the ability to conduct an association study. In a separate study involving a large number of ASD patients and controls, 102 diverse genes were identified as risk factors for ASD through exome-wide association analysis. Notably, only *OFD1*, *KMT2C*, and *PTEN*—the genes highlighted in the current study—appeared in this association study [57]. This discrepancy between gene lists may be due to patient- or population-based heterogeneity and methodological differences. Therefore, additional association studies involving a larger Turkish patient cohort and control group are necessary.

## 5. Conclusions

In our study, we analyzed WES results from 75 Turkish patients diagnosed with ASD and identified disease-causing variants in 24% of these patients. Such research underscores the role of WES in diagnosing ASD at the molecular level. Although detection rates may differ among ethnic and clinical groups and cohort sizes, ongoing gene discovery is likely to improve these rates. The identified patterns of pathogenic variants could also aid in discovering new variants, enriching current knowledge. Additionally, this information helps medical geneticists classify variants more accurately for individual patients. One limitation is that parental data was not available for all VUSs. Further patient data and functional research are needed to validate these findings, especially to clarify the pathogenicity of VUSs.

## Figures and Tables

**Table 1:** List of pathogenic or LP variants in the presence of demographic information of the patients.

Patient No.	Age (y/o)	Gender	Genetic Variants	Rs/Allele Frequency in GnomAD Global	P or LP (ACMG)	Zygosity	Inheritance	Disease with Diagnosis (#OMIM), Inheritance	Phenotype
P2	3.5	Male	*BRWD3* (NM_153252.5):c.2800C>T:p.Arg934Ter	-/Not reported	LP (PVS1, PM2)	Hem	Maternal	Intellectual developmental disorder, X-linked 93 (300659), XLR	ASD, DD, relatively macrocephaly
P3	10	Female	*CAMK2A* (NM_015981.4):c.310C>T:p.Arg104Trp	-/Not reported	LP (PM2, PS2, PP2, PP3, PP5)	Het	De novo	Intellectual developmental disorder, autosomal dominant 53 (617798), AD	ASD, congenital bilateral hearing loss
P4	3	Male	*EP300* (NM_001429.4):c.6568C>T:p.Gln2190Ter	-/Not reported	LP (PVS1, PM2)	Het	ND	Menke-Hennekam syndrome 2 (6183339), AD	ASD
P5	6	Male	*EP300* (NM_001429.4):c.256C>T:p.Arg86Ter	rs1601598122/ Not reported	LP (PVS1, PS4, PM2, PP4)	Het	ND	Rubinstein–Taybi syndrome 2 (613684), AD	ASD, DD, microcephaly (−5 SD), short stature (−2.75 SD), beaked nose, broad thumbs and toes *
P6	4	Male	*KMT2C* (NM_170606.3):c.6650C>T:p.Pro2217Leu	-/Not reported	LP (PS2, PM2)	Het	De novo	Kleefstra syndrome 2 (617768), AD	ASD
P8	10	Female	*PTEN* (NM_000314.8):c.202T>C:p.Tyr68His	rs398123317/ Not reported	P (PS4, PS3, PM2, PM5_P, PP2, PP3)	Het	Paternal	Macrocephaly-autism syndrome (605309), AD	ASD, ID
P9	9	Male	*PTEN* (NM_000314.8):c.142A>G:p.Asn48Asp	-/Not reported	LP (PS2, PM2, PM5, PP2, PP3)	Het	De novo	Macrocephaly-autism syndrome (605309), AD	ASD, macrocephaly (4.6 SD)
P10	4	Male	*SMG9* (NM_019108.4):c.897del:p.Tyr299Ter	rs765635618/ 3.9 × 10^−6^	LP (PVS1, PM2)	Het	Paternal	Neurodevelopmental disorder with intention tremor, pyramidal signs, dyspraxia, and ocular anomalies (619995), AR	ASD, intermittent exotropia
			*SMG9* (NM_019108.4):c.898G>A:p.Val300Ile	rs768806823/ 2.4 × 10^−5^	LP (PM2, PM3, PP3, PP4)	Het	Maternal		
P52	16	Male	*SHANK3* (NM_001372044.2):c.3286_3287delinsT:p.Ala1096SerfsTer44	-/Not reported	LP (PVS1, PM2)	Het	ND	Phelan–McDermid syndrome (606232), AD	ASD
P53	10	Male	*MECP2* (NM_001110792.2):c.1174_1192del: p.Val392CysfsTer23	-/Not reported	LP (PVS1_M, PM2, PP5_M)	Hem	ND	Intellectual developmental disorder, X-linked, syndromic 13 (300055), XLR	ASD, drug resistant epilepsy
P54	19	Male	*MECP2* (NM_001110792.2):c.524G>A:p.Gly175Glu	-/Not reported	LP (PS2, PM2, PM5_P, PP3)	Hem	De novo	Intellectual developmental disorder, X-linked, syndromic 13 (300055), XLR	ASD, DD, epilepsy, microcephaly (−3.8 SD)
P55	8	Female	*MECP2* (NM_001110792.2):c.916C>T:p.Arg306Ter	rs61751362/ Not reported	P (PVS1, PS2, PS3, PM2)	Het	De novo	Rett syndrome (312750), XLD Rett syndrome, atypical (312750), XLD	ASD, developmental regression, epilepsy
P58	14	Female	*PPP2R5D* (NM_006245.4):c.598G>A:p.Glu200Lys	rs863225079/ Not reported	P (PS3, PS4, PM2, PP2, PP3)	Het	ND	Houge-Janssens syndrome 1 (616355), AD	ASD, ID, epilepsy, absence of speech
P59	18	Male	*SCN1A* (NM_001165963.4):c.3733C>T:p.Arg1245Ter	rs727504136/ Not reported	P (PVS1, PS4, PM2)	Het	De novo	Dravet syndrome (607208), AD	ASD, epilepsy
P60	10	Male	*ZDHHC9* (NM_016032.4):c.777+1G>A	-/Not reported	P (PVS1, PM2, PP5)	Hem	Maternal	Intellectual developmental disorder, X-linked syndromic, Raymond type (300799), XL	ASD, DD, dysmorphic appearance
P61	13	Male	*TRRAP* (NM_001375524.1):c.2879G>A:p.Arg960Gln	-/Not reported	LP (PS2, PM2, PP2)	Het	De novo	Developmental delay with or without dysmorphic facies and autism (618454), AD	ASD, ID, microcephaly (−2.7 SD)
P62	9	Male	*UBE3A* (NM_130839.5):c.1021C>T:p.Gln341Ter	rs587781191/ Not reported	P (PVS1, PS4, PM2)	Het	De novo	Angelman syndrome (105830), AD	ASD, ID, atrial septal defect
P71	7	Male	*OFD1* (NM_003611.3):c.2883C>A:p.Tyr961Ter	-/Not reported	LP (PM1, PM2, PM4, PP3, PP4)	Hem	ND	*OFD1*-related ciliopathy, XLR	ASD, speech delay, postaxial polydactyly. There was no MRI scan.

AD = autosomal dominant, AR = autosomal recessive, ASD = autism spectrum disorder, DD = developmental delay, Het = heterozygous, ND = not determined, ID = intellectual disability, Hem = hemizygous, P = pathogenic, LP = likely pathogenic, XLR = X-linked recessive, XLD = X-linked dominant, * In this patient, a 22q11.2 duplication was also detected in the chromosomal microarray analysis.

**Table 2:** List of variants of unknown significance in the presence of demographic information of the patients.

Patient No.	Age (y/o)	Gender	Genetic Variants	Rs/Allele frequency in gnomAD global	Zygosity	Inheritance	Gene-related diseases (#OMIM), Inheritance	Phenotype
P7	4	Male	*GRIN2B* (NM_000834.5):c.3619G>A:p.Glu1207Lys	-/Not reported	Het	ND	Intellectual developmental disorder, autosomal dominant 6, with or without seizures (613970), AD	ASD, speech delay
P11	16	Male	*SYN1* (NM_006950.3):c.2055C>G:p.Asp685Glu	-/Not reported	Hem	ND	Epilepsy, X-linked 1, with variable learning disabilities and behavior disorders (300491), XLR	ASD, macrocephaly (+2 SD)
P12	8	Male	*MED13L* (NM_015335.5):c.2423A>G:p.His808Arg	-/Not reported	Het	ND	Impaired intellectual development and distinctive facial features with or without cardiac defects (616789), AD	ASD
P13	18	Male	*DIP2B* (NM_173602.3):c.2620A>G:p.Ser874Gly	-/Not reported	Het	ND	Intellectual developmental disorder, autosomal dominant, FRA12A type (136630), AD	ASD
			*PPP2R1A* (NM_014225.6):c.740C>G:p.Thr247Ser	-/Not reported	Het	ND	Houge-Janssens syndrome 2 (616362), AD	
P17	10	Male	*RALGAPA1* (NM_001346249.2):c.1097C>G:p.Ser366Cys	rs921047919/ Not reported	Hom	Paternal or maternal	Neurodevelopmental disorder with hypotonia, neonatal respiratory insufficiency, and thermodysregulation (618797), AR	ASD, DD
P19	6	Male	*BRWD3* (NM_153252.5):c.3326-4C>G	-/Not reported	Hem	ND	Intellectual developmental disorder, X-linked 93 (300659), XLR	ASD, DD
P22	6	Male	*CNOT1* (NM_016284.5):c.3068_3079del:p.Gln1023_Val1026del	-/Not reported	Het	ND	Vissers-Bodmer syndrome (619033), AD	ASD, speech delay
			*FBXO11* (NM_001190274.2):c.935-7T>A	-/Not reported	Het	ND	Intellectual developmental disorder with dysmorphic facies and behavioral abnormalities (618089), AD	
			*ZBTB7A* (NM_015898.4):c.839C>G:p.Ala280Gly	-/Not reported	Het	ND	Macrocephaly, neurodevelopmental delay, lymphoid hyperplasia, and persistent fetal hemoglobin (619769), AD	
P24	2	Male	*DEAF1* (NM_021008.4):c.62C>G:p.Ala21Gly	-/Not reported	Het	ND	Vulto-van Silfout-de Vries syndrome (615828), AD	ASD
P25	7	Male	*SMARCA1* (NM_001282874.2):c.837G>A:p.Met279Ile	-/Not reported	Hem	ND	X-linked neurodevelopmental disorders, XL	ASD, DD
			*KDM6B* (NM_001348716.2):c.3302A>G:p.Lys1101Arg	-/Not reported	Het	ND	Stolerman neurodevelopmental syndrome (618505), AD	
P26	3	Male	*MED13L* (NM_015335.5):c.5365C>A:p.Arg1789Arg ^1^	-/Not reported	Het	ND	Impaired intellectual development and distinctive facial features with or without cardiac defects (616789), AD	ASD
P30	6	Male	*KIF1A* (NM_001244008.2):c.1564G>A:p.Asp522Asn	-/Not reported	Het	ND	NESCAV syndrome (614255), AD	ASD, speech delay
P31	4	Female	*CDH15* (NM_004933.3):c.795C>T:p.Phe265Phe ^2^	rs1220962174/ Not reported	Het	ND	Intellectual developmental disorder, autosomal dominant 3 (612580), AD	ASD, delayed myelination
P32	6	Female	*ANKRD17* (NM_032217.5):c.6343G>T:p.Val2115Phe	-/Not reported	Het	ND	Chopra–Amiel–Gordon syndrome (619504), AD	ASD
P33	5	Male	*PRORP* (NM_014672.4):c.1202G>A:p.Arg401His	rs547075844/ 1.1 × 10^−5^	Hom	ND	Combined oxidative phosphorylation deficiency 54 (619737), AR	ASD
P34	3	Female	*GRIK2* (NM_021956.5):c.1117G>A:p.Gly373Ser	rs914040945/ Not reported	Het	ND	Neurodevelopmental disorder with impaired language and ataxia and with or without seizures (619580), AD	ASD
			*SRCAP* (NM_006662.3):c.3751A>T:p.Ile1251Phe	-/Not reported	Het	ND	Developmental delay, hypotonia, musculoskeletal defects, and behavioral abnormalities (619595), AD	
P35	3	Female	*SHANK1* (NM_016148.5):c.3625C>T:p.Pro1209Ser	-/Not reported	Het	ND	Neurodevelopmental disorder, AD	ASD
P36	3	Female	*GRIN2A* (NM_001134407.3)c.676G>C:p.Val226Leu	-/Not reported	Het	ND	Epilepsy, focal, with speech disorder and with or without impaired intellectual development (245570), AD	ASD
P37	10	Male	*TAF4* (NM_003185.4):c.2563A>G:p.Thr855Ala	-/Not reported	Het	ND	Intellectual developmental disorder, autosomal dominant 73 (620450), AD	ASD
P38	13	Male	*BPTF* (NM_182641.4):c.4588C>G:p.Gln1530Glu	-/Not reported	Het	ND	Neurodevelopmental disorder with dysmorphic facies and distal limb anomalies (617755), AD	ASD, ADHD
			*TCF20* (NM_001378418.1):c.3782T>G:p.Ile1261Ser	-/Not reported	Het	ND	Developmental delay with variable intellectual impairment and behavioral abnormalities (618430), AD	
P39	4	Male	*GRIN2B* (NM_000834.5):c.1262G>A:p.Ser421Asn	-/Not reported	Het	ND	Developmental and epileptic encephalopathy 27 (616139), AD Intellectual developmental disorder, autosomal dominant 6, with or without seizures (613970), AD	ASD
P40	5	Female	*EHMT1* (NM_024757.5):c.974G>T:p.Gly325Val	-/Not reported	Het	ND	Kleefstra syndrome 1 (610253), AD	ASD, speech delay
			*DIP2B* (NM_173602.3):c.904G>A:p.Glu302Lys	-/Not reported	Het	ND	Intellectual developmental disorder, autosomal dominant, FRA12A type (136630), AD	
P45	6	Female	*JMJD1C* (NM_032776.3):c.1640C>A:p.Ser547Tyr	-/Not reported	Het	Maternal	Intellectual disability (PMID: 26181491)	ASD
P56	16	Male	*PHIP* (NM_017934.7):c.4801T>C:p.Ser1601Pro	-/Not reported	Het	ND	Chung–Jansen syndrome (617991), AD	ASD, resistant epilepsy
P57	7	Female	*PPP2CA* (NM_002715.4):c.879_880del:p.Arg294Ter	-/Not reported	Het	ND	Houge-Janssens syndrome 3 (618354), AD	ASD, ID, DD, epilepsy
			*COL4A3BP* (NM_001379029.1):c.852_854delinsGAA:p.Arg285Lys	-/Not reported	Het	ND	Neurodevelopmental disorder with hypotonia, speech delay, and dysmorphic facies (616351), AD	
P63	12	Male	*POGZ* (NM_015100.4):c.-1-3T>G	-/Not reported	Het	ND	White–Sutton syndrome (616364), AD	ASD, epilepsy
P64	15	Female	*SETD2* (NM_014159.7):c.6997G>A:p.Gly2333Arg	rs1559661037/ Not reported	Het	ND	Intellectual developmental disorder, autosomal dominant 70 (620157), AD Luscan–Lumish syndrome (616831), AD Rabin-Pappas syndrome (620155), AD	ASD, epilepsy, dysmorphic findings
P66	8	Male	*HERC2* (NM_004667.6):c.11669G>T:p.Arg3890Ile	-/Not reported	Het	Paternal	Intellectual developmental disorder, autosomal recessive 38 (615516), AR	ASD, epilepsy, learning difficulties
			*HERC2* (NM_004667.6):c.1150G>A:p.Glu384Lys	rs772532798/ 8.8 × 10^−5^	Het	ND		
P67	4.5	Female	*KMT2B* (NM_014727.3):c.2318A>G:p.Glu773Gly	-/Not reported	Het	Maternal (affected)	Intellectual developmental disorder, autosomal dominant 68 (619934), AD	ASD, DD
			*CDK8* (NM_001260.3):c.402G>T:p.Gln134His	-/Not reported	Het	Maternal (affected)	Intellectual developmental disorder with hypotonia and behavioral abnormalities (618748), AD	
			*SRCAP* (NM_006662.3):c.5144C>G:p.Thr1715Arg	-/Not reported	Het	ND	Developmental delay, hypotonia, musculoskeletal defects, and behavioral abnormalities (619595), AD	
P69	4	Male	*TCF20* (NM_001378418.1):c.2513A>C:p.Asp838Ala	-/Not reported	Het	ND	Developmental delay with variable intellectual impairment and behavioral abnormalities (618430), AD	ASD, DD
P70	3	Female	*TNRC6B* (NM_001162501.2):c.1553A>G:p.Asp518Gly	-/Not reported	Het	ND	Global developmental delay with speech and behavioral abnormalities (619243), AD	ASD, bicuspid aortic valve, aortic dilatation
P72	14	Female	*CEP41* (NM_018718.3):c.1079G>C:p.Arg360Pro	rs781878740/ 3.9 × 10^−6^	Hom	ND	Joubert syndrome 15 (614464), AR	ASD, DD, microcephaly (−5.8 SD), spasticity, visual impairment, bilateral frontotemporal atrophy on MRI (3.5-year-old)
P73	9	Male	*GRIN1* (NM_007327.4):c.1668G>C:p.Gln556His	-/Not reported	Het	ND	Neurodevelopmental disorder with or without hyperkinetic movements and seizures (614254), AD	ASD, epilepsy, DD, hippocampal atrophy, micropenis, puberty tarda
P74	11	Male	*KMT2B* (NM_014727.3):c.7160-4C>T	-/Not reported	Het	ND	Intellectual developmental disorder, autosomal dominant 68 (619934), AD	ASD, obesity (+4 SD), tall stature (+3.92 SD), precocious puberty, hypothyroidism
P75	7	Male	*C12ORF57* (NM_138425.4):c.35G>T:p.Ser12Ile	rs148483779/ 8.8 × 10^−4^	Hom	Paternal or maternal	Temtamy syndrome (218340), AR	ASD, DD, EEG abnormalities, congenital glaucoma *

AD = autosomal dominant, AR = autosomal recessive, ADHD = attention deficit hyperactivity disorder, ASD = autism spectrum disorder, DD = developmental delay, EEG = electroencephalogram, Hem = hemizygous, Het = heterozygous, Hom = homozygous, ID = intellectual disability, ND = not determined, XLR = X-linked recessive. ^1^ First base of the exon. ^2^ Third base of the exon. * In this patient, compound heterozygous pathogenic variants were detected in the *CYP1B1* gene associated with glaucoma findings.

## Data Availability

The datasets analyzed during the current study are available from the corresponding author upon reasonable request.
